# Dilemmas in the Choice of Adequate Therapeutic Treatment in Patients with Acute Pulmonary Embolism—From Modern Recommendations to Clinical Application

**DOI:** 10.3390/ph15091146

**Published:** 2022-09-14

**Authors:** Ratko Lasica, Milika Asanin, Lazar Djukanovic, Nebojsa Radovanovic, Lidija Savic, Marija Polovina, Sanja Stankovic, Arsen Ristic, Marija Zdravkovic, Andjelka Lasica, Jelena Kravic, Jovan Perunicic

**Affiliations:** 1 Department of Cardiology, Emergency Center, University Clinical Center of Serbia, 11000 Belgrade, Serbia; 2 Center for Medical Biochemistry, University Clinical Center of Serbia, 11000 Belgrade, Serbia; 3 Faculty of Medical Sciences, University of Kragujevac, 34000 Kragujevac, Serbia; 4 Department of Cardiology, University Clinical Center of Serbia, 11000 Belgrade, Serbia; 5 Clinical Center Bezanijska Kosa, 11000 Belgrade, Serbia; 6 Health Center New Belgrade, 11000 Belgrade, Serbia

**Keywords:** acute pulmonary embolism, dilemmas, therapeutic treatment, recommendations, clinical application

## Abstract

Pulmonary thromboembolism is a very common cardiovascular disease, with a high mortality rate. Despite the clear guidelines, this disease still represents a great challenge both in diagnosis and treatment. The heterogeneous clinical picture, often without pathognomonic signs and symptoms, represents a huge differential diagnostic problem even for experienced doctors. The decisions surrounding this therapeutic regimen also represent a major dilemma in the group of patients who are hemodynamically stable at initial presentation and have signs of right ventricular (RV) dysfunction proven by echocardiography and positive biomarker values (pulmonary embolism of intermediate–high risk). Studies have shown conflicting results about the benefit of using fibrinolytic therapy in this group of patients until hemodynamic decompensation, due to the risk of major bleeding. The latest recommendations give preference to new oral anticoagulants (NOACs) compared to vitamin K antagonists (VKA), except for certain categories of patients (patients with antiphospholipid syndrome, mechanical valves, pregnancy). When using oral anticoagulant therapy, special attention should be paid to drug–drug interactions, which can lead to many complications, even to the death of the patient. Special population groups such as pregnant women, obese patients, patients with antiphospholipid syndrome and the incidence of cancer represent a great therapeutic challenge in the application of anticoagulant therapy. In these patients, not only must the effectiveness of the drugs be taken into account, but great attention must be paid to their safety and possible side effects, which is why a multidisciplinary approach is emphasized in order to provide the best therapeutic option.

## 1. From Diagnostic Doubts to the Correct Diagnosis of Pulmonary Embolism

Venous thromboembolism (VTE) is the third most common cardiovascular disease (after myocardial infarction and cerebrovascular insult), with an estimated annual incidence in epidemiological studies of 1–2 cases per 1000 people [1,2]. The frequency of VTE largely depends on age, gender, and associated diseases.

Most episodes of VTE are provoked by the presence of a number of risk factors, and in some episodes of VTE it is not possible to establish a clear risk factor (unprovoked PE) [3].

Major traumatic injuries, surgery, lower extremity fractures, knee or hip arthroplasty, myocardial infarction (within the previous three months), previous VTE, as well as spinal cord injury are **strong risk factors**. Blood transfusions, the use of drugs that stimulate erythropoiesis, chemotherapy, autoimmune diseases, thrombophilia (Factor V Leiden gene mutation, activated protein C resistance, prothrombin gene mutation (G 20210 A), AT3 deficiency, protein C and S deficiency, elevated values of lipoprotein), the presence of a central venous catheter, heart failure, stroke, the postpartum period, as well as infections are classified as **moderate risk factors** [4]. Malignant diseases comprise a well-known predisposing factor for VTE. It has been shown that 20% of patients with VTE have an active malignant disease [5]. It has been shown that cancer patients have an increased risk of recurrence of PE, major bleeding, and risk of early and three-month mortality after PE [6]. The use of oral contraceptives is the most common risk factor in women during the reproductive period [7]. **Weak risk factors** for the development of PE are older age, diabetes, hypertension, obesity, varicose veins, pregnancy, laparoscopic surgery, immobilization for more than 3 days, and prolonged sitting [4]. A more recent division of risk factors for PE includes four groups: **major transitory** (traumas, major surgical interventions); **major persistent** (malignant diseases and antiphospholipid syndrome); **minor transient** (oral contraceptives, pregnancy, puerperium); **minor persistent** (congenital thrombophilias, autoimmune diseases). Strong transient risk factors are responsible for approximately 20% of all VTE episodes [8].

The diagnosis of this sometimes insidious disease is also complicated by the fact that the specificity and sensitivity of symptoms associated with PE is very low, so the presence or absence of any symptom is not enough to confirm or exclude the existence of PE [9]. The most common symptoms and signs of PE are dyspnea (30–88%) [10,11,12], a pleuritic type of chest pain (from 39–70%) [9,10,13], leg swelling, which is suspicious for deep vein thrombosis (DVT) (24%) [14], hemoptysis (2–11.8%) [15,16], cough (9–23%) [12,16] syncope (6–39%) [9,10,16], tachycardia (40%) [9,10,11], hypoxemia (70%) [17,18], and new-onset atrial fibrillation (24%) [8].

In recent times, the tendency to reduce the unnecessary costs of testing patients with suspected PE on the one hand and overlooking non-specific signs and symptoms of the disease on the other hand are the causes of the largest number of missed diagnoses of PE. By standardizing algorithms for the diagnosis and treatment of patients with suspected PE, the diagnostic accuracy of this disease has been improved [19]. These diagnostic algorithms include pre-test probability assessments for the existence of PE, the evaluation of D-dimer values as well as non-invasive radiographic techniques. However, in clinical practice, the non-specific clinical picture of PE represents a great challenge for a diagnosis of this disease. The initial presentation of patients with PE varies from asymptomatic cases (discovered incidentally during a diagnostic examination to detect other diseases) to high-risk PE resulting in RV dysfunction and the subsequent development of shock. The symptoms of the disease in PE can vary and gain in dynamics. Thus, patients initially misdiagnosed with PE may deteriorate clinically to such an extent that re-evaluation will lead to a definitive diagnosis of PE. On the other hand, patients who are hemodynamically unstable with cardiogenic shock may raise the suspicion of an initially cardiac cause of such a condition.

It is recommended that in patients with a low or moderate pre-test probability for PE, D dimer values are also determined, while in patients with a high probability, sophisticated diagnostic methods are immediately implemented [4,20]. However, both decreased and elevated D dimer values can lead the doctor to misdiagnose cases. Elevated D-dimer values are also found in patients with inflammatory processes, who suffer from chronic renal failure, cancer, during pregnancy, injuries, and surgeries [21,22]. Patients with elevated D-dimer levels due to their low positive predictive value should undergo further diagnostic testing to confirm and/or rule out the diagnosis of PE. Given that normal D-dimer values increase with age, it is necessary to take the patient’s age into account when interpreting the results, which is achieved with age-adjusted D-dimer values [23]. Studies have shown that an age-adjusted D-dimer cutoff may be effective and safe in ruling out PE compared with conventional D-dimer (500 µg/L) [24,25]. Elevated values of the D-dimer are physiologically present in pregnancy (they increase in relation to the trimester of pregnancy) which can lead to a wrong diagnosis [26]. Clinical suspicion of the existence of PE and elevated D dimer values are often the reason for performing CT pulmonary angiography and/or ventilation–perfusion scanning, which exposes both the mother and the fetus to the harmful effects of radiation [27]. In the case of negative D-dimer values combined with a low clinical probability, PE can be ruled out without further diagnostic procedures [20]. A study that analyzed data from the national collaborative study (PIOPED II) showed that despite a low clinical probability for the presence of PE (according to the Wells score), patients had a CT-proven embolus in the main or lobar branches of the PA [13].

Despite advances in medicine, Alonso-Martínez et al. showed that misdiagnosis occurred in 50% (95% CI 44–55) of patients. A higher age, more days of delay and the absence of syncope or sudden-onset dyspnea were factors associated with misdiagnosis [28]. To reduce the number of missed diagnoses of PE, the YEARS rule can be used, which consists of three items: the clinical suspicion of PE, presence of clinical signs of DVT, and the presence of hemoptysis [4]. The Pulmonary Embolism Rule-out Criteria (PERC) score is also used, where it is possible to rule out PE in patients with a low clinical probability who, in addition, have fulfilled all eight criteria of the PERC rule. The impossibility of its widespread use is reflected in the fact that it can only be applied in clinical settings with a low (<5%) prevalence of PE [4,17]. Tome van der Hulle and colleagues showed in a prospective cohort study that D-dimer testing in combination with clinical pretest probability assessments using the YEARS criteria or the revised Geneva score can exclude pulmonary embolism [29].

In a systematic review by Kwok Chun S and associates (analysis of 18 studies), it was shown that the most common diagnoses with which PE is mixed are pneumonia, bronchitis, exacerbation of chronic obstructive pulmonary disease, heart failure and acute coronary syndrome [30].

The presence of individual signs and symptoms for PE has a low sensitivity and specificity, while combining them increases the probability that a patient suspected of PE really has this disease. The latest European recommendations for the diagnosis of PE suggest the use of the revised Geneva score and the Wells score. Regardless of which score is used, the expected percentage of patients with PE is about 10% in the low-probability category, 30% in the intermediate category, and 65% in the high-probability category [4].

Although changes in the electrocardiogram (ECG) in patients with PE are considered insufficiently specific and sensitive for establishing a diagnosis, they can point us to this disease. Most often, sinus tachycardia can be registered in the ECG (present in about 40% of patients); sign S1Q3T3 (Mc Ginn White sign—in about 10% of patients); complete or incomplete right bundle branch block (present in about 25%; in hemodynamically unstable patients up to 30%) [31,32]. The presence of right bundle branch block in the electrocardiogram in hemodynamically stable patients is often associated with RV dysfunction compared to patients without it (15% vs. 5%, *p* < 0.001) [33]. Right bundle branch block in patients with PE is associated with a poor prognosis [34]. Ermis et al. also found an association between presence of right axis deviation and the severity of PE with this finding in 3% of low-risk PE, 15% of intermediate-risk PE and 28% of high-risk PE cases (*p* = 0.009) [35]. P pulmonale occurs in the ECG in up to 19% with acute PE. ST segment depression in leads V1–V6 is present in about 26% of PE patients. The presence of inversion T waves in anterior leads has been reported with variable frequency from 16% to 68% [32]. The presence of atrial fibrillation in patients with PE was observed in 15% to 21% of patients [36]. The Qr configuration in V1 (Weber and Phillips sign) is specific for PE but has a low prevalence (11–19%) [37]. This sign is a predictor of RV dysfunction.

**Echocardiography** is a non-invasive method with a huge role in the diagnosis and clinical assessment of patients with PE. Enlargement of RV, hypokinesia of the free wall of RV and interventricular septal flattening were found in 27.4%, 26.6%, and 18.4% of patients, respectively [38]. An enlarged RV with akinesia of the basal segment of the free wall (McConnell’s sign) can be seen in approximately 20% of patients with PE [38]. The presence of this sign has 77% sensitivity and 94% specificity for the diagnosis of acute PE [39]. Casazza et al. demonstrated that McConnell’s sign can also be seen in cases of RV infarction and thus cannot be considered pathognomonic for acute PE [40,41]. The only sure sign for the existence of PE with this method is the visualization of thrombus masses in the right heart cavities (Supplementary Materials, Figures S1 and S2), which occurs in 4–18% of patients with acute PE [42]. **Transesophageal echocardiography**, when it comes to central PE, has a sensitivity of 90–95% and a specificity of 100%. Thromboembolus is very difficult to visualize in the middle part of the left pulmonary artery because the interposition of the left main bronchus interferes with the ultrasound beam (Supplementary Materials, Figures S3 and S4; Video S1).

Thanks to the results of The Prospective Investigation On Pulmonary Embolism Diagnosis (PIOPED) II study, **CT pulmonary angiography (CTPA)** has become the method of choice for the diagnosis of PE (sensitivity of 83% and specificity of 96%) [13] (Supplementary Materials, Figures S5–S7).

## 2. Risk Stratification in Patients with PE

Risk stratification in patients with acute PE is necessary to determine the initial therapeutic approach. Determining the correct initial therapeutic regimen is very important because the risk of early mortality in normotensive patients is still high and amounts to 2–8%, while in patients presenting with cardiogenic shock it is up to 30%, and in the case of the need for cardiopulmonary resuscitation, the risk of early mortality increases up to 65% [43]. Initial stratification is based on clinical symptoms and signs: hemodynamic status at initial presentation, PESI score (Pulmonary Embolism Severity Index) and simplified PESI score (sPESI), and the presence of RV dysfunction based on transthoracic echocardiography results and biomarker values (Troponin and BNP-a) [4,44]. Cardiac troponin, a marker of myocardial damage, was shown to be a significant predictor of early mortality in patients with PE, and the presence of positive troponin even in the case of low risk, as assessed by the PESI score, indicates higher early mortality [45]. RV pressure overload due to PE is associated with increased myocardial distension causing the release of BNP and N-terminal pro BNP (NT pro BNP). Natriuretic peptide levels reflect the severity of RV dysfunction in acute PE [46].

**Figure 1:**
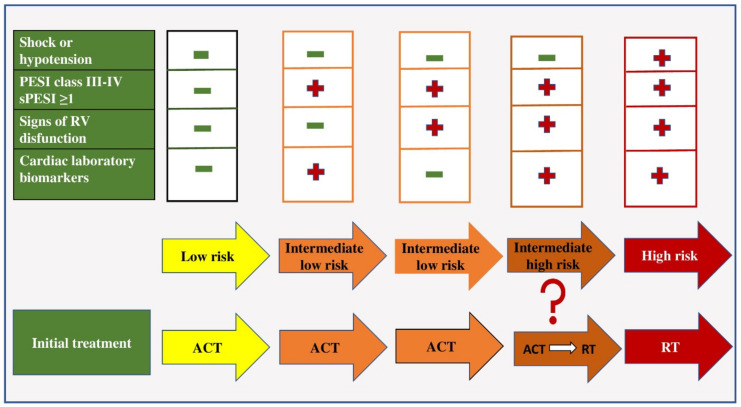
Early mortality risk and initial treatment in patients with acute pulmonary embolism. Legend: **ACT**—anticoagulant therapy; **RT**-reperfusion therapy; **PESI**—The Pulmonary Embolism Severity Index Score; **sPESI**—simplified Pulmonary Embolism Severity Index Score.

All patients with PE can be classified into three risk categories: low, intermediate and high. Patients who are hypotensive (systolic blood pressure less than 90 mmHg) or in shock (systolic blood pressure less than 80 mmHg) on admission belong to the category of patients with high-risk PE (about 5% of PE cases) [4,47]. Such patients should be treated with thrombolytic therapy and/or embolectomy (Figure 1).

In the group of patients in whom hemodynamic instability is not present, further risk stratification is based on the assessment of prognostic criteria, namely clinical, visualization and laboratory indicators (mostly related to proving the presence of RV dysfunction). Patients with a low risk of early mortality are normotensive on admission, have a sPESI score of less than one, have no RV dysfunction on echocardiographic examination and have negative biomarker values and are treated with anticoagulant therapy [48].

Patients with an intermediate risk of early mortality are hemodynamically stable on admission and have an sPESI score greater than one. If they have positive values or biomarkers or signs of RV dysfunction proven by echocardiography, they belong to the group of patients with intermediate–low risk and they should be treated with anticoagulant therapy [4]. If these patients have positive biomarker values and the presence of RV dysfunction, they belong to the intermediate–high risk group and are initially treated with anticoagulant therapy, and in case of hemodynamic decompensation, with thrombolytic therapy [49].

It is also important to note that the risk of pulmonary embolism is a dynamic category, so the initially set therapeutic decision can be changed depending on the patient’s clinical condition. Choosing the right initial treatment for PE patients not only affects their survival, but also reduces the frequency of post-thromboembolic pulmonary hypertension, which worsens the long-term prognosis of these patients and reduces their quality of life.

## 3. Initial Therapeutic Regimen in the Acute Phase of PE

### 3.1. Anticoagulant Therapy

The initiation of anticoagulant therapy without delay is necessary in patients with a high and intermediate clinical probability for PE, while the diagnostic procedure is still in progress. Hemodynamically stable patients should be treated with anticoagulant therapy: unfractionated heparin (UFH), low-molecular-weight heparin (LMWH) or new oral anticoagulants (NOACs: apixaban, dabigatran, edoxaban or rivaroxaban) [50]. NOACs are administered depending on whether oral therapy can be administered, depending on kidney function and other circumstances. Parenteral anticoagulant therapy is based on the use of indirect and direct anticoagulant drugs. The activity of indirect anticoagulant drugs is based on binding to plasma cofactors, while direct anticoagulant drugs do not need a plasma cofactor to exert their effect. The subcutaneous administration of low-molecular-weight heparin or fondoparinux or the intravenous administration of unfractionated heparin is common.

#### 3.1.1. Unfractionated Heparin

Unfractionated heparin is an indirect anticoagulant that achieves its effect by binding to antithrombin III. The resulting complex inactivates thrombin and factors Xa, IXa, XIa and XIIa [51]. It also inhibits the aggregation of platelets, activates osteoclasts, and inhibits the formation of osteoblasts [52]. It achieves its anticoagulant effect by reducing the propagation of thrombus and preventing new embolic events. The main limitation of heparin stems from its tendency to bind to positively charged plasma proteins, proteins released from platelets and endothelial cells, which can result in a variable anticoagulant response and the phenomenon of heparin resistance [53,54]. Due to its largely dose-dependent clearance, the plasma half-life of UFH ranges from 30–60 min. [51,54]. One of the side effects of heparin is the occurrence of heparin-induced thrombocytopenia (HIT). The frequency of HIT ranges from 1 to 5% of patients receiving heparin [55]. Type I is the most common form of thrombocytopenia and is self-limiting [56]. Mild thrombocytopenia occurs in the first 2 days after heparin initiation and normalizes even with continued heparin therapy [55]. A more serious form (HIT, Type II) is an immune-mediated disorder characterized by the formation of antibodies against the heparin–PF4 complex. Typically, the platelet count declines by >50% between days 5 and 14 of heparin treatment [57]. When compared with low-molecular-weight heparin (LMWH), UFH is much more likely to cause HIT [58,59]. Prandoni P et al. showed in a prospective study that the incidence of HIT in patients who received LMWH was about 0.8%. It was also shown that patients were more likely to get HIT if they were treated with UFH before LMWH (1.7 vs. 0.3%, OR, 4.9; 95% CI, 1.5–16) [60]. By activating osteoclasts, heparin can affect the occurrence of osteopenia. The initial dose of UFH depends on body weight and is 80 IU/kg in an intravenous bolus, and then continues with 18 IU/kg intravenous in a continuous infusion [4]. The therapeutic effect of heparin is controlled by determining the activated partial thromboplastin time (aPTT), which should be maintained in the range of 1.5 to 2.5 times higher than the starting aPTT.

The use of UFH is currently mostly limited to patients with obvious hemodynamic instability or immediate hemodynamic decompensation, in whom primary reperfusion treatment will be necessary. Intravenous unfractionated heparin is also recommended for patients with severe renal impairment (creatinine clearance < 30 mL/min) and/or severe obesity [4,61].

In case of HIT development, it is necessary to use direct thrombin inhibitors (lepirudin, argatroban, bivalirudin) as well as factor Xa inhibitors (eg fondaparinux).

#### 3.1.2. Low-Molecular-Weight Heparin

Low-molecular-weight heparin—its anticoagulant action is mostly achieved by inhibiting activated factor X. LMWH advantages over heparin in that it has a better bioavailability and longer half-life, simplified dosing, predictable anticoagulant response, lower risk of HIT, and lower risk of osteoporosis [61]. LMWH is administered in fixed doses. The administration of LMWH does not require aPTT monitoring and can be administered subcutaneously [62,63]. The usual dose of LMWH for therapeutic anticoagulation is 1 mg/kg given every 12 h, although another option is 1.5 mg/kg given once daily for VTE. The dose efficacy of LMWH should also be monitored in patients with renal dysfunction, as LMWH is excreted by the kidneys, so dose adjustments are sometimes necessary in relation to creatinine clearance. Unlike UFH, the clearance of LMWH is dose-independent, and the half-time in plasma is approximately 4 h. AntiXa level monitoring is not routinely recommended, but its therapeutic range is 0.6–1.0 mmol/L. The following LMWHs are approved for the treatment of PE: enoxaparin, tinzaparin, dalteparin, and nadroparin [4].

The main complication during UFH and LMWH treatment is bleeding. In patients treated for VTE, a lower risk of bleeding was registered when using LMWH compared to UFH (OR = 0.68, *p* = 0.05). In the Systematic Review of the Cochrane Collaboration on VTE, LMWH was shown to be significantly safer than UFH (incidence of major bleeding, 1% vs. 2.1%, OR 0.57; 95% CI 0.39–0.83) [62]. The advantage of intravenous administration of UFH is that the overdose can be easily neutralized by the administration of protamine sulfate. Protamine is packaged as an intravenous solution in a concentration of 10 milligrams/milliliter and its maximum dose is 50 mg. Protamine sulfate in a dose of 1 mg neutralizes 100 IU of UFH [64]. Although protamine sulfate can be used as an antidote to LMWH, it does not completely neutralize the anticoagulant activity of LMWH because it binds only to the longer chains of LMWH [65]. One milligram of protamine sulfate will neutralize approximately 100 anti-Xa units of a LMWH [66]. Similar to UFH, protamine dosing for LMWH reversal is dependent on the timing of LMWH administration relative to the need for reversal [66]. Taking that into consideration, if reversal is necessary within 8 h of receiving a LMWH, a full dose of protamine should be administered. In patients with elevated LMWH anti-Xa assays 2 to 4 h after protamine administration, a second dose of protamine to achieve complete reversal may be considered. If a second dose is given, it is recommended to administer 0.5 mg of protamine per 1 mg of enoxaparin or 100 units of dalteparin [66].

For both enoxaparin and dalteparin, protamine does not provide a complete reversal of anti-Xa activity. It is estimated that protamine will reverse up to about 60% to 75% of anti-Xa activity.

#### 3.1.3. Fondaparinux

Fondaparinux is also a factor Xa inhibitor. Its half-life is 17 h. Because fondaparinux is cleared unchanged via the kidney, it is contraindicated in patients with a creatinine clearance below 30 mL/min and should be used with caution in those with a creatinine clearance below 50 mL/min. [67]. Unlike LMWH, there is no cross-reactivity between Fondaparinux and HIT antibodies [68]. Lori-Ann Linkins et al., within the framework of a systematic review conducted on nine studies, concluded that the use of Fondaparinux is effective and safe for the treatment of HIT, although according to the recommendations, this drug is not included for the treatment of this condition [69]. The major side effect of fondaparinux is bleeding. There is no antidote for this drug. An anticoagulant effect is achieved when administered subcutaneously in a dose of 2–8 mg, or intravenously in a dose of 2 mg to 20 mg. Due to its almost complete bioavailability after subcutaneous administration, as well as its long half-life and lack of variability in anticoagulant response, fondoparinux can be administered in a single daily dose without laboratory monitoring [70]. In the treatment of DVT and PE, it is given in a dose of 7.5 mg for patients with a body weight of 50–100 kg, 5 mg for patients under 50 kg and 10 mg for patients over 100 kg body weight [4]. Fondoparinux does not bind to protamine sulfate. If bleeding occurs, recombinant factor VII may be effective [71].

#### 3.1.4. Direct Thrombin Inhibitors

Direct thrombin inhibitors are used in patients who develop HIT. Direct thrombin inhibitors are a class of anticoagulants that inhibit thrombin generation by binding to the active site on thrombin. They are classified as univalent or bivalent inhibitors. Univalent inhibitors such as argatroban interact with the active, catalytic site of thrombin itself, compared to bivalent inhibitors (hirudin and its derivatives), which recognize the binding site on fibrinogen. Three prospective studies, HAT-1, HAT-2 and HAT-3, have demonstrated the effectiveness of **lepirudin** as an anticoagulant in reducing the risk of thromboembolic complications in patients with HIT [72,73,74]. The pharmacodynamic effect of lepirudin on thrombin activity was assessed using the aPTT. The prolongation of aPTT was linear over the range of plasma concentrations tested (up to 0.5 mg/kg i.v.). Lepirudin can be successfully used subcutaneously in patients with HIT. The mean half-life was around 1.7 h. The dose was shown to correlate linearly with aPTT response with lepirudin doses of 0.75–2 mg/kg s.c. in two daily doses. The elimination of lepirudin is predominantly through the kidneys, so in patients with renal failure, the half-time of elimination can be significantly prolonged, so dose adjustment is recommended [75]. The recommended dose of lepirudin in patients with normal renal functions is a 0.4 mg/kg i.v. push (over 15–20 s), followed by continuous infusion at 0.15 mg/(kg/h). **Argatroban** has a half-life of 40–50 min and requires dose reduction both in patients with liver damage and in critically ill patients. For the prophylaxis or treatment of thrombosis in HIT, the recommended starting dose of argatroban is 2 µg/kg/min for patients without hepatic impairment [76]. The starting dose should be reduced in patients with moderate hepatic impairment to 0.5 mg/kg/min. The initial dose can be adjusted up to 10 mg/kg/min to achieve a stable aPTT 1.5–3 times longer than basal. **Bivalirudin** is also a direct thrombin inhibitor that is injected at a dose of 0.05–0.1 mg/kg/h. Argatroban may be advantageous when compared to bivalirudin in achieving initial therapeutic anticoagulation goals among patients with suspected or confirmed HIT [77]. The American Society of Hematology 2018 guidelines for management of venous thromboembolism: heparin-induced thrombocytopenia suggests the possible use of NOACs in HIT [78]. With respect to the choice of NOAC, most of the published experience in HIT is with rivaroxaban [79] (Table 1).

### 3.2. Thrombolytic Therapy

The application of thrombolytic therapy, systemic or directed by a catheter into the pulmonary artery, is used in patients with high-risk PE, is accompanied by hemodynamic instability [47,80]. Thrombolytic therapy in these patients leads to recanalization of the blood vessel and normalization of the hemodynamic status, which is accompanied by a reduction in RV dilatation on echocardiography. Thrombolytic drugs dissolve blood clots by activating plasminogen, which forms a product called plasmin. Plasmin is a proteolytic enzyme capable of breaking the cross-links between fibrin molecules, which ensure the structural integrity of blood clots. The greatest benefit is seen if therapy is started within the first 48 h of symptom onset, although thrombolysis may also be beneficial in patients who have had symptoms for 6–14 days [47,80]. Medicines that are used from this group are: streptokinase, urokinase and recombinant tissue plasminogen activator (rtPA) [4]. Contraindications to the use of thrombolytic therapy can be divided into absolute (history of hemorrhagic stroke, ischemic stroke in the last 6 months, neoplasm of the central nervous system, major trauma or surgery, head injury in the previous 3 weeks, hemorrhagic diathesis, active bleeding) and relative (transient ischemic attack in the previous 6 months, use of oral anticoagulant drugs, pregnancy, refractory hypertension, advanced liver disease, infective endocarditis, active peptic ulcer) [81]. The potential benefits are often complicated by the relatively high incidence of hemorrhagic complications. Within the meta-analysis of Saurav Chatterjee et al. the frequency of major bleeding during the use of thrombolytic therapy was shown to be 9.24% [82].

### 3.3. Embolectomy

#### 3.3.1. Surgical Embolectomy

Surgical embolectomy with cardiopulmonary bypass can be performed in patients with acute PE accompanied by hemodynamic instability and a contraindication to thrombolytic therapy [49]. Lee T et al. did not show a significant difference in 30 day mortality when using medical thrombolytic therapy and surgical embolectomy (15 vs. 13% respectively) [83]. Stein et al. reported that the mortality of pulmonary embolectomy for acute PE was 20% in a meta-analysis of 1300 cases [84]. Case series have also been published that have shown variable results with perioperative mortality ranging from 2.3% to 27.2% [85]. Extracorporeal membrane oxygenation (ECMO) in combination with surgical embolectomy can be used in case of hemodynamic instability of the patient with contraindications for the use of thrombolytic therapy [86].

#### 3.3.2. Percutaneous Catheter Embolectomy

Percutaneous catheter embolectomy is the removal of a thrombus from the pulmonary circulation and involves: (1) fragmentation of the thrombus using a balloon catheter or loop; (2) rheolytic thrombectomy using hydrodynamic catheters; (3) suction thrombectomy using aspiration catheters and (4) rotary thrombectomy [87]. For patients who do not have absolute contraindications to thrombolytic treatment, percutaneous catheter embolectomy or pharmacomechanical thrombolysis is preferred [4].

### 3.4. Initial Therapeutic Regimen in Patients with Intermediate–High-Risk Pulmonary Embolism

Patients with an intermediate–high-risk of PE are a very heterogeneous group of patients, in whom there is still a dilemma regarding the overall therapeutic benefit of using fibrinolytic therapy compared to heparin treatment. According to study data: Moderate pulmonary embolism treated with thrombolysis (MOPET); Management Strategies and Prognosis of Pulmonary Embolism-3 (MAPPET-3); Treatment of submassive pulmonary embolism with tenecteplase or placebo: Cardiopulmonary Outcomes at Three months (TOPCOAT); Fibrinolysis for Patients with Intermediate-Risk Pulmonary Embolism (PEIHTO): patients with moderate–high risk PE had a significant clinical benefit from the application of thrombolytic therapy [88,89,90]. In the PEIHTO study in patients with intermediate–high risk PE, fibrinolytic therapy with anticoagulant therapy compared to placebo prevented death and hemodynamic decompensation of the patients (2.6% vs. 5.6% respectively) but increased major bleeding (6.3 vs. 1.2%) and stroke [90]. A meta-analysis by Saurav Chatterjee et al. showed that in intermediate-risk pulmonary embolism trials, thrombolysis was associated with a lower mortality (OR, 0.48; 95% CI, 0.25–0.92) and more major bleeding events (OR, 3.19; 95% CI, 2.07–4.92) when compared with anticoagulant therapy [82]. A meta-analysis by Nakamuras et al. showed that thrombolytic therapy significantly reduced the incidence of the composite endpoint of all-cause death or clinical deterioration (3.9% vs. 9.4%; RR, 0.44; *p* < 0.001) [91]. For now, the decision to apply thrombolytic therapy is left to the prescribing physician, who should summarize the risk of clinical deterioration (age, comorbidities, degree of RV dysfunction, biomarker values, respiratory status) and the risk of hemorrhagic complications. In addition, different combinations of clinical and laboratory parameters were used to construct prognostic scores, which allow for the semiquantitative assessment of the early risk of death associated with PE. One of those risk scores is the Bova score, which includes: RV dysfunction, arterial blood pressure values, heart rate, and troponin values. The model identified three stages (I, II and III) with 30-day PE-related complication rates of 4.2%, 10.8% and 29.2%, respectively [92]. In 2021, the CHEST recommendations and the report of the expert panel on the indications for the use of thrombolytic therapy in patients with moderate-risk PE were published [93]. These recommendations also suggest that hemodynamically stable patients be treated aggressively with anticoagulant and not thrombolytic therapy with careful monitoring, and that only if hemodynamic decompensation occurs should thrombolysis be administered. Apart from hypotension, other types of deterioration such as a progressive increase in heart rate, drop in systolic blood pressure (>90 mm Hg), decrease in saturation or partial pressure of oxygen in arterial blood, worsening of RV dysfunction and increase in biomarker values are indications for the use of thrombolytic therapy. For a long time, the question was raised as to what dose of thrombolytics would be effective in these patients without increasing the hemorrhagic risk. Results of a randomized pilot study by Wang et al. in 2010 showed that the administration of half the therapeutic dose of rtPA (50 mg) in patients who are hemodynamically triggered is not inferior to the full dose in terms of RV function recovery [94]. However, the results of the aforementioned study are not supported by current guidelines for the treatment of intermediate–high risk PE [4].

## 4. Prolonged Treatment with Anticoagulant Therapy of Patients with PE

The development of awareness that PE is often recurrent has led to a more precise consideration of the duration of anticoagulant therapy after the initial event. According to the current recommendations of the European Association of Cardiologists for the diagnosis and treatment of PE, extended treatment with anticoagulant therapy should last at least three months, and in a large number of patients, longer than three months [4]. The continuation of anticoagulant therapy for longer than 3–6 months leads to a reduction of 80% or more in the occurrence of recurrent VTE, but an increase in the risk of major bleeding by one to three times with NOACs and four to five times with vitamin K antagonists [95]. Patients with VTE who are associated with transient risk factors have a low recurrence risk of 3%. In this group of patients, oral anticoagulant therapy for three or more months is justified (assuming that the risk factor has been resolved). Patients with idiopathic (unprovoked) thromboembolism have a significantly higher recurrence rate, corresponding to an annual event rate of 7.9%. In these patients, the prolonged use of anticoagulant therapy should last longer than 3 months [96]. Oral anticoagulant treatment of indefinite duration is recommended for patients who have recurrent VTE not associated with a reversible risk factor. In these patients, the interval of re-evaluation of the continuation of oral anticoagulant therapy must be determined, depending on the risk/benefit ratio. Patients who have any of the thrombophilias (Factor V Leidem mutation, MTHFR mutation and prothrombin 20210 mutation) are candidates for the prolonged use of anticoagulant therapy. Patients with antiphospholipid syndrome, active cancer, and protein S, C, and antithrombin III deficiency are at significant risk for thromboembolic recurrence and lifelong anticoagulation should be considered [97]. Aspirin is not recommended for the prolonged treatment of PE, given its lower efficacy and similar safety profile when compared to NOACs [98]. In all patients who had a first episode of VTE, it is important to assess the risk of repeated episodes of thromboembolism. This can be done using several different scores such as the Vienna prediction model (VPM), DASH score and HERDOO2 scoring system, which use different patient parameters such as gender, D-dimer values, and location of the thrombus during the first event [99,100]. The significance of these scores is that they can help clinicians decide on the duration of NOACs after the first VTE.

### 4.1. Vitamin K Antagonists

For more than 60 years, vitamin K antagonists were the only oral anticoagulants available for clinical use. Vitamin K antagonists are rapidly absorbed from the gut and 90% are bound to circulating albumin. The half-life of VKA is 36 to 48 h, and hepatic metabolism by microsomal enzymes, including cytochrome P450 variants, partially explains the marked interindividual variation in the doses required for drug effect. A number of factors influence the biological activity of VKA, including the patient’s age, patient’s activity level, diet, drug absorption, albumin levels, vitamin K intake, general health status, and use of various medications.

Drug–drug interactions are very important when it comes to the safety of drug administration, especially in patients with multiple comorbidities, who are treated with a large number of drugs. Cholestyramine and sucralfate are thought to reduce the gastrointestinal absorption of warfarin [101]. On the other hand, drugs such as amiodarone, fluvastatin, lovastatin, fluconazole and sertaline can potentiate the effect of warfarin by inhibiting CYP2C9 [101]. By binding to plasma proteins, drugs such as ibuprofen, quinidine, fenofibrate, losartan, valsartan, amlodipine and felodipine can take over the important site needed for warfarin metabolism and thus potentiate its anticoagulant effect [102]. Metronidazole, fluconazole, trimethoprim–sulfamethoxazole, and miconazole can affect the effect of warfarin by inhibiting CYP 1A or CYP 3A4, and macrolide antibiotics by inhibiting CYP3A4 [102]. The use of antibiotics can reduce the absorption of vitamin K by affecting the intestinal flora [103]. Concomitant use of antiplatelet drugs such as aspirin and P2Y12 receptor inhibitors with warfarin greatly increases the risk of bleeding [104]. The interaction of warfarin with selective serotonin reuptake inhibitors (SSRIs) leads to a decrease in serotonin in platelets and a decrease in platelet aggregation [105]. Nonsteroidal anti-inflammatory drugs (NSAIDs) affect the activity of warfarin by inhibiting cytochrome p450, and by inhibiting the digestion of prostaglandins, they can lead to the formation of erosions on the stomach, increasing the risk of gastrointestinal bleeding [106]. The most recent meta-analysis showed a higher degree of clinically relevant bleeding with the combined use of warfarin and antiplatelet drugs (OR = 1.74; 95% CI 1.56, 1.94), antibiotics (OR = 1.63; 95% CI 1.45, 1.83), NSAIDs (OR = 1.83; 95 % CI 1.29, 2.59), and SSRIs (OR = 1.62; 95% CI 1.42, 1.85), compared with warfarin alone [107]. Additionally, within this meta-analysis, no increase in clinically relevant bleeding was demonstrated with the combined use of amiodarone, a beta blocker. In this study, no drug combined with warfarin had a significant effect on thromboembolic events or mortality. Many statins (fluvastatin, lovastatin, simvastatin, atorvastatin) bind to plasma proteins and affect the prolongation of the INR value in patients with concomitant use of these drugs [108,109]. This is confirmed by a study by Anna E and collaborators who measured the INR values in patients who used warfarin with some of the statins (simvastatin, rosuvastatin, atorvastatin). Simvastatin initiation led to an increase in mean INR from 2.40 to 2.71, with INRs peaking after 4 weeks, corresponding to a mean change of 0.32 (95% CI 0.25–0.38). The initiation of atorvastatin and rosuvastatin led to INR increases of 0.27 (95% CI 0.12–0.42) and 0.30 (95% CI −0.09–0.69) [110].

Many studies have shown that various types of supplements and herbs can affect CYP2C9 activity, so caution should always be exercised in their combined use with warfarin [111]. When it comes to supplements, it has been shown that the combined intake of grapefruit, mango, ginkgo biloba, and cranberry with warfarin can prolong the INR and increase the likelihood of bleeding, while the use of St. John’s wort and green tea can reduce its effect [112,113,114,115,116,117]. The concomitant consumption of foods rich in vitamin K (lettuce, broccoli, spinach, green peas) was associated with a risk of reduced therapeutic effect of VKA [112].

The varying effectiveness of the drug is expressed in its varying INR. Because of occasionally high INR values, patients are exposed to the risk of hemorrhagic complications, and because of low INR values, to the risk of thromboembolic events. The risk of bleeding is directly related to the INR; it begins to rise quite significantly when the INR increases to more than 3.5, and an INR greater than 5 requires the administration of fresh frozen plasma or vitamin K [118]. The average oral dose of warfarin is 5 mg/day, and for patients over 70 years old it is 4 mg/day. The optimal target INR for the treatment and prophylaxis of VTE is 2.0–3.0. Given the long half-life of both VKA (36 h) and factor II (60 h), to achieve a full anticoagulant effect the INR must be administered in the therapeutic range for at least 4 to 6 days to achieve a 20–30% reduction in vitamin K-dependent coagulation factors compared to normal [4]. Frequent INR controls, constant INR variations and the risk of thrombosis/bleeding have made NOACs preferable except in specific indications (antiphospholipid syndrome, presence of mechanical valves, extremely reduced renal function, severe mitral stenosis).

### 4.2. New Oral Anticoagulant Drugs

In recent years, novel treatment strategies with direct oral anticoagulants (NOACs) have been increasing in popularity and availability. NOACs are preferred over vitamin K antagonist (VKA) therapy. This is due to a similar reduction in the risk of recurrent VTE, a reduced risk of bleeding and easier monitoring of their effect compared to vitamin K antagonists [119]. Each of the NOACs showed similar results compared to VKA when it comes to recurrent PE [120,121]. In a meta-analysis by Mohammad Alhousani and colleagues, NOACs and other anticoagulants (VKA and LMWH) showed no statistical difference in preventing recurrent VTEs among patients with chronic renal failure, but NOACs had a significantly lower risk of major and non-major clinically relevant bleeding regardless of the level of renal impairment when compared to VKAs [122].

#### 4.2.1. Apixaban

The oral bioavailability is approximately 50%, with most of the drug absorption occurring in the small intestine [123]. Drug elimination occurring via the metabolism through the CYP3A4 systems in the intestine and liver and the P-glycoprotein system can be enhanced through drug–drug interactions with apixaban [124]. Apixaban is given at a dose of 10 mg twice a day for 7 days and then 5 mg twice a day [4]. Andexanet alfa can be used to reverse apixaban (off-label) in life-threatening or uncontrollable bleeding. The dosage is based on the specific factor Xa agent-inhibitor to be reversed, the dose, and the time since the last dose was administered [124]. An andexanet alfa 400 mg intravenous bolus is administered at a rate of 30 mg/min, followed by 4 mg/min via continuous infusion for up to 120 min, to reverse apixaban (5 mg or less) or rivaroxaban (10 mg or less), administered within 8 h or if the time is unknown [124].

#### 4.2.2. Rivaroxaban

Rivaroxaban is an oral direct factor Xa inhibitor approved for the prevention and treatment of DVT and PE [4]. Rivaroxaban is given in a dose of 2 × 15 mg for 3 weeks and then 20 mg once a day. Renal elimination accounts for approximately 36% of the unchanged drug. Its use in patients with a CrCL < 30 mL/min is not advised, and <15 mL/min employment is contraindicated [125]. Rivaroxaban is not dialyzable. A significant increase in rivaroxaban exposure was demonstrated with the strong CYP3A4 inhibitors, ketoconazole (158% increase (95% CI 136%, 182%) for a 400 mg once daily dose) and ritonavir (153% increase (95% CI 134%, 174%)). Therefore, the use of rivaroxaban should be avoided with strong combined CYP3A4 inhibitors (mainly antifungals (except fluconazole) and human immunodeficiency virus (HIV) protease inhibitors) [126].

#### 4.2.3. Dabigatran

Dabigatran is a direct thrombin (IIa) inhibitor approved for the prevention and treatment of DVT and PE [4]. Caution must be used in the elderly, as the risk of stroke and bleeding increases with age, as seen in an analysis of the RE-LY (Randomized Evaluation of Long-Term Anticoagulant Therapy) trial [127]. Dabigatran is contraindicated for its use in patients with mechanical heart valves. The RE-ALIGN (Randomized, Phase II Study to Evaluate the Safety and Pharmacokinetics of oral Dabigatran Etexilate in Patients after Heart Valve replacement) trial was terminated early due to thromboembolic events (valve thrombosis, stroke, and myocardial infarction), and major bleeding was observed in the dabigatran group compared to the warfarin group in heart valve patients [124,128]. Dabigatran is a substrate for P-glycoprotein 1 (P-gp) and its concomitant use with strong P-gp inhibitors such as verapamil and ketoconazole or P-gp inducers such as rifampicin, carbamazepine and phenytoin may affect its effect [102]. Although the co-administration of dabigatran with most Pgp inhibitors does not require dose adjustment, it should be administered at least 2 h before these drugs when co-administered. In the documents of the US Food and Drug Administration, verapamil increases the exposure, i.e., the effectiveness, of dabigatran. The European Medicines Agency recommendations and clinical guidance suggest a dabigatran dose reduction to 110 mg when given with verapamil regardless of kidney function. No dose adjustment is needed with the concomitant use of P-gp inhibitor amiodarone [129]. The use of dabigatran with ketoconazole and itraconazole should be avoided because its bioavailability increases with their simultaneous use [130]. Care should also be taken when using St. John’s wort and dabigatran because their simultaneous use may affect the reduced activity of the drug. No interaction was observed when dabigatran was coadministered with atorvastatin, an HMG-CoA reductase inhibitor. When the granules are taken without a protective capsule, their bioavailability increases by as much as 75% when compared to an intact capsule [131]. Therefore, it is not advisable to crush or chew the medicine. Due to the fact that the capsules are designed to be released in the stomach, they must not be used in patients receiving medication through a nasogastric or jeunostomy tube. It is mainly eliminated by the kidneys and is the only NOAC that can be removed by hemodialysis [132].

Dabigatran is given in a dose of 150 mg twice a day and for patients older than 80 years in a dose of 110 mg twice a day. The agent named idarucizumab is a monoclonal antibody with 350 times more affinity for dabigatran than thrombin.

#### 4.2.4. Edoxaban

Approval was based primarily on the Hokusai VTE study, which evaluated 3319 patients with PE. The trial showed that edoxaban was not inferior to warfarin but had a lower bleeding risk [133]. The oral bioavailability is approximately 60%, and renal elimination accounts for approximately 50% of unchanged drug. Edoxaban is given 60 mg once a day and 30 mg once a day if the body weight is ≤60 kg. The simultaneous use of edoxaban and ritonavir, erythromycin, azithromycin, clarithromycin, and ketoconazole should be avoided due to a significant increase in its plasma concentration [102,134]. If macrolide antibiotics and edoxaban are necessary, its dose can be reduced by 50% [135]. Amiodarone may increase edoxaban exposure by 40%. In the case of cyclosporine and tacrolimus administration, dose adjustment is necessary [136]. A study by Mainbourg S and associates examined the use of NOACs (apixaban, edoxaban, rivaroxaban and dabigatran) in different dosage regimens and analyzed whether the dosage method correlates with the occurrence of thrombogenic events. There was no difference in major thrombotic events (RR BID/QD = 1.06, 95% IC 0.86–1.30) nor in major bleeding (RR BID/QD = 1.02, 95% IC 0.84–1.23) between NOACs that were dosed twice daily or once a day, without heterogeneity (I2 = 0%) [137]. If oral anticoagulation continues after 3 months of PE in a patient without cancer, after 6 months of therapy, consider reducing the NOAC dose: apixaban (2.5 mg daily) or rivaroxaban (10 mg daily). When using all NOACAs, the use of proton-pump inhibitors (PPIs) is also suggested. No interactions between PPIs, apixaban, rivaroxaban and edoxaban have been shown. Administration of dabigatran and PPIs reduced the absorption of dabigatran by 20%, because an acidic environment is necessary for the absorption of dabigatran.

#### 4.2.5. Inhibitori Aktiviranog Faktora XIa

Newer anticoagulant drugs that inhibit activated coagulation factor XI could potentially reduce thromboembolic events while reducing the risk of major bleeding. In the analysis of PuY C. et al., it is stated that patients with factor XI deficiency would have a lower incidence of VTE stroke in the general population [138]. Recently, the results of the PACIFIC-AF study were published, comparing the safety of an activated factor XI inhibitor (asundexian used in two doses—20 mg or 50 mg) with a NOAC (apixaban—5 mg in two daily doses) in patients with AF and an increased risk both from stroke and bleeding. In a Phase II trial, PACIFIC-AF showed a significant reduction in significant bleeding with asundexian, an oral factor XIa inhibitor, in comparison to apixaban [139].

The weight of the decision to discontinue anticoagulant therapy in patients who need to undergo a surgical procedure is reflected in the possibility of an increased risk of thromboembolism due to the discontinuation of anticoagulant therapy, and an increased risk of bleeding due to the intervention itself in patients who are on anticoagulant therapy [140]. In the case of a high risk of bleeding, which can be assessed based on the type of surgery and associated comorbidities, a longer pause in the use of anticoagulant therapy is required. In the case of surgical interventions with a low risk of bleeding, their performance sometimes does not require the suspension of anticoagulant drugs [141]. In the event of the need for elective surgery within the first three months after PE, postponing of it is advised. In the case of the need for urgent surgical intervention in the early period after PE, bridging NOACs with LMWH can be used to reduce the interval without anticoagulants. Warfarin is usually stopped five days before the elective surgery with the INR value being checked [142]. In the event that the INR value is >1.5, a dose of oral vitamin K (1–2 mg) can be administered to normalize the INR value and achieve a value of ≤1.4 [142]. It takes four to six days for the INR to return to normal after stopping warfarin. For surgery with a low/moderate risk of bleeding, it is recommended that NOAC therapy be omitted one day before and continued for one day (approximately 24 h) after the procedure, provided hemostasis is secure [141,143]. The total duration of the interruption is two days. For surgery with a high bleeding risk, NOACs should be omitted two days before and continued for two days (approximately 48 h) after the procedure, provided hemostasis is secure. The total duration of the interruption is four days. For individuals with impaired renal function (creatinine clearance (CrCl) <30 to 50 mL/min) taking dabigatran, there is an additional one-day withdrawal before low/moderate-bleeding-risk procedures and an additional two-day withdrawal before high-bleeding-risk procedures [141].

## 5. Treatment of PE in Specific Patient Populations

### 5.1. Treatment of PE in Pregnancy

Acute PE remains one of the leading causes of maternal death. The risk of VTE is higher in pregnant women compared to women of a similar age who are not pregnant. Given that they do not cross the placental barrier, the use of UFH and LMWH has proven to be the safest during pregnancy [45]. Due to the ease of administration and known pharmacodynamics, LMWHs are used most often. In pregnant women in whom PE occurs a month before delivery, it is possible to schedule induced delivery with the suspension of LMWH 24 h before delivery. According to ESC recommendations in high-risk situations, for it is recommended that LMWH be converted to UFH ≥ 36 h prior to delivery [4]. The UFH infusion should be stopped 4–6 h prior to anticipated delivery [45]. The use of VKA therapies is not recommended for pregnant women considering that they pass through the placenta and their use is associated with embryopathies, fetal and neonatal bleeding [144]. Cases of placental abruption are also reported during their use. The use of NOAC therapy is contraindicated in pregnant women [4]. Data on the use of NOACs in pregnancy indicate an increased rate of miscarriage (31%) compared to the rate of miscarriage in the general population. Bone and facial abnormalities in the fetus were observed in 4% of pregnancies during rivaroxaban use, although as many as 85% of women discontinued NOACs within the first two months of pregnancy [145]. The decision to use anticoagulants during pregnancy, especially before delivery, requires a multidisciplinary team of anesthesiologists, gynecologists and cardiologists in order to reduce unwanted hemorrhagic side effects. After delivery, anticoagulant treatment in lactating women includes the use of UFH, LMWH, vitamin K antagonists and fondaparinux. NOACs are concentrated in breast milk and are contraindicated but may be considered in women who are not breastfeeding, or after breastfeeding in those who have an indication for longer-term treatment.

### 5.2. Treatment of PE in Antiphospholipid Syndrome

Antiphospholipid syndrome is associated with a high risk of recurrent venous thrombosis. The persistent presence of elevated values of antiphospholipid antibodies during the first manifestation of VTE is an indication for an unlimited duration of anticoagulant therapy. Recommendations of the European Association of Cardiologists do not support the use of NOACs instead of VKA [4]. According to a study by Vittorio Pengo et al., the use of rivaroxaban was associated with an increased rate of thromboembolic and major bleeding events when compared with warfarin [146]. Such data were confirmed by the I Trial of Rivaroxaban in AntiPhospholipid Syndrome (TRAPS) study. Rivaroxaban was compared with warfarin in patients with high-risk antiphospholipid syndrome. Data from this study support earlier studies on the superiority of warfarin over rivaroxaban in high-risk patients with antiphospholipid syndrome [147]. Given that the majority of studies examined the effect of NOACs in patients with high-risk antiphospholipid syndrome, further studies could examine the effectiveness in lower-risk antiphospholipid syndrome and thus identify special groups of patients in whom NOAC therapy would be efficient.

### 5.3. Treatment of PE in Patients with Cancer

In patients with cancer, the annual frequency of the first VTE differs depending on the type of cancer (3% in patients with bladder and breast cancer; 4–7% in patients with colon and prostate cancer; 10–12% in lung, stomach, ovarian cancer and brain; 15% in pancreatic cancer) [17]. Cancer patients have a four to seven times higher risk of VTE, three times the risk of recurrent VTE, two times the risk of bleeding when using anticoagulants, and ten times the risk of death when compared to non-cancer patients. The presence of comorbidities in cancer patients, such as anemia, kidney failure, and reduced mobility can further complicate the treatment of these patients. The first line of therapy for the treatment of PE in patients with cancer is LMWH, and among the NOACs, apixaban and rivaroxaban are also approved for the treatment of these patients. NOACs have shown non-inferiority in reducing VTE recurrence compared to LMWH in the most recent meta-analysis and randomized controlled trials [148]. However, for edoxaban and dabigatran, a high bleeding risk was found when compared to LMWH, especially for patients with gastrointestinal tract tumors [149,150]. Within the TacDOAC registry, the occurrence of thrombosis and bleeding in patients with antitumor therapy who received NOACE was analyzed. The incidence of major bleeding ranged from 2.3% to 9.5%, and the clinically relevant rates of minor bleeding ranged from 2.3% to 14.3%. Within this registry, many of the anticancer drugs used were weak or moderate CYP3A4 inhibitors with or without P-gp inhibitory properties [151,152]. Caution is required when using even mild inducers/inhibitors of CYP3A4/P-gp, especially in the case of polypharmacy or in the presence of more than two bleeding risk factors. Among anticancer medications, the tyrosin kinase inhibitors strongly affect the P-gp activity, and imatinib, crizotinib, vandetanib and sunitinib are contraindicated with NOAC-s [141].

On the other hand, the use of monoclonal antibodies is associated with a lower risk of interactions with NOACs, although the use of alemtuzumab is contraindicated with all NOACS, while bevacizumab (anti VEGF), caplacizumab (anti-vWF), ipilimumab (anti CTLA4) and ramucirumab (anti-VEGFR2) could be used with caution in the presence of NOACs [153]. The use of doxorubicin and vinblastine can affect the reduction of NOAC levels in plasma, so their simultaneous use is not recommended [141]. When it comes to immunomodulatory agents such as cyclosporine and tacrolimus, their use is not indicated with dabigatran [153]. Tacrolimus should also be avoided with other NOACs, and cyclosporine in combination with edoxaban [141].

The SELECT-D study compared the rate of recurrent VTE and the risk of bleeding in cancer patients depending on whether they were treated with rivaroxaban or dalteparin. Rivaroxaban was found to be associated with a relatively low VTE recurrence but higher clinically relevant minor bleeding when compared with dalteparin [154]. However, the latest evidence on apixaban has suggested that apixaban is the best alternative, among NOACs, in patients with gastrointestinal cancers due to a lower risk of major bleeding [155]. The ADAM VTE trial [107], which randomized 300 patients to apixaban or LMWH for six months, reported higher recurrent thrombosis in the LMWH group, without any difference in the safety outcomes of major bleeding or clinically relevant bleeding [156].

However, in the treatment of VTE in cancer patients who are treated with a large number of drugs, it should not be forgotten that the simultaneous administration of NOACs and drugs that can affect CYP3A4 and/or P-gp can lead to significant interactions [157]. For these reasons, LMWHs remains the treatment of choice in these patients because their metabolism does not involve either CYP3A4 or P-gp [4].

### 5.4. Treatment of PE in Elderly and Frail Patients

The age of the patient represents not only the risk of VTE but also of bleeding, especially in frail patients, in whom numerous pathophysiological changes lead to changes in drug kinetics and possible toxicity even in standard doses of oral anticoagulants [158]. Frailty is a medical syndrome characterized by a progressive decline in homeostatic and physiological reserves [159]. A recent meta-analysis examined the safety and efficacy of NOACs compared to VKA in patients with acute VTE over 70 years of age. The primary safety endpoint of major or clinically relevant non-major bleeding was significantly reduced in NOACs as compared to VKAs in both patients with age < 75 years (OR: 0.79, 95% CI: 0.70–0.89) and patients with an age of more than 75 years (OR: 0.75, 95% CI: 0.59–0.96) [160].

NOACs were associated with a reduced risk of major bleeding in elderly patients compared to warfarin (relative risk ratio 0.39, 95% CI 0.17–0.90). The results of a meta-analysis examining the efficacy and safety of NOACs compared to VKAs in elderly patients with AF showed greater safety and efficacy in the NOACa group, while apixaban was the best among NOACs when compared to warfarin (HR, 0, 64; 95% CI: 0.33–1.30) [161]. The possibility of using lower doses of NOACs in this population should be considered in view of the presence of many comorbidities [162].

### 5.5. Treatment of PE in Patients with Renal Failure

When treating patients with PE who have a certain degree of renal weakness, it must be taken into account that all four available NOACs have a partial route of elimination through the kidneys, with dabigatran having the highest degree of renal elimination (80%) (edoxaban—50%; rivaroxaban—33%; apixaban—22%) [158]. The results of a meta-analysis examining the efficacy and safety of NOACs in the elderly with impaired renal function showed no significant difference in efficacy between NOACs and warfarin (OR, 0.71, 95% CI 0.41–1.21) [163]. Additionally, in the more recent meta-analysis by Su X. et al. in the treatment of acute VTE, NOACs did not significantly reduce recurrent VTE or VTE-related death (OR, 0.96; 95% CI, 0.82 to 1.11) but significantly reduced bleeding events (0.76, 0.68 to 0.90) when compared with warfarin [164]. A small number of studies examined the use of NOACs in patients with severely impaired renal function and patients on hemodialysis. A study by Chan KE et al. found an increase in the risk of hospitalization or death due to bleeding with dabigatran (RR, 1.48; 95% CI 1.21–1.81; *p* = 0.0001) and rivaroxaban (RR, 1.38; 95% CI 1.03–1.83; *p* = 0.04) compared to warfarin in the HD population, suggesting that these two drugs are not entirely safe in HD patients [165]. It is possible that apixaban administration is the safest in patients with renal impairment due to the lowest degree of renal elimination.

In all patients who are planned to be introduced to NOACs, it is necessary to initially assess renal function. Also, in patients treated with NOACs, it is very important to monitor changes in renal function at least once a year in order to correct the dose of anticoagulant drugs in a timely manner.

### 5.6. Treatment of PE in Obese Patients

Although the guidelines (ISTH SSC) from 2016 did not advise the use of NOACs in extremely obese people (body mass index [BMI] > 40 kg/m^2^ or weight > 120 kg), new recommendations from 2021 suggest that rivaroxaban and apixaban can be adequate for the treatment of VTE in patients with obesity regardless of body weight and BMI [166]. In a meta-analysis by Mohamed Nabil Elshafei et al., which was conducted on five observational studies with 6585 patients with severe obesity (BMI ≥ 40 kg/m^2^ or weight ≥ 120 kg), NOACs (rivaroxaban, apixaban, and dabigatran) were compared with warfarin for the treatment of VTE. The authors found a similar efficacy of NOACs (recurrent VTE OR 1.07; 0.93–1.23) and a nonsignificant trend toward a reduced risk of major bleeding (OR 0.80; 0.54–1.17) [167]. According to a post hoc analysis of the EINSTEIN study including 861 patients with a BMI ≥ 35 kg/m^2^ there was no significant difference in recurrent VTE in those using rivaroxaban compared with warfarin at even 21 days (2.1% vs. 0.9%, respectively; HR 2.22; 0.68–7.26), nor after 12 months (3% vs. 2.1%, respectively; HR 1.45; 0.62–3.39). There were no differences in the occurrence of major bleeding in the studied groups [168].

### 5.7. Treatment of PE in HIV Patients

With the development and application of modern, highly active antiretroviral therapy (HAART), the survival of patients with HIV infection has increased. HART is defined as any treatment regimen consisting of three or more drugs with different mechanisms of action (protease inhibitor (PIs), nonnucleoside reverse transcriptase inhibitor (NNRTIs), nucleoside reverse transcriptase inhibitor (NRTIs), integrase strand transfer inhibitor, CCR5 antagonist, fusion inhibitor, pharmacokinetic booster) [169]. Most drugs from the group of PIs affect the activity of CYP3A4 [170]. Ritonavir is the most potent CYP3A4 inhibitor and, for this reason, is often combined in low doses with other protease inhibitors to produce a “booster” effect of the protease inhibitor without significantly increasing side effects. However, in addition to acting via CYP3A4, ritonavir is also a strong P-gp inhibitor and therefore may dually increase the exposure to NOACs [171]. The European Heart Rhythm Association Practical Guide on the Use of Non-Vitamin K Antagonist Oral Anticoagulants in Patients with Atrial Fibrillation from 2021 does not recommend the use of HIV protease inhibitors in combination with NOACs [141]. These recommendations advise that in the case of the combined use of NOACs and antiretroviral therapy in HIV patients, NOAC dose reduction should be considered—although this approach should be limited to centers with extensive clinical experience.

Similarly, the pharmacoenhancer cobicistat (a potent CYP3A4 inhibitor) is contraindicated for use with NOACs [171]. According to some authors, NOACs could be used with darunavir and tipranavir with caution and regular monitoring [169]. The theoretical advantage in the selection of NOACs in HIV patients could be given to dabigatran, considering that it is not subject to metabolism via CYP3A4, but there are no clinical studies to confirm this [172]. On the other hand, antiretroviral drugs acting on P-gp can affect the activity of dabigatran, considering that it is a substrate for P-gp. When Pgp inhibitors were administered concurrently, the bioavailability of dabigatran was increased by up to 200% [170]. Most NNRTIs are inducers of CYP3A4; therefore, the use of efavirenz and etravirine is not recommended because they can potentially affect the reduction of apixaban and rivaroxaban concentrations [169]. Etavirine is a weak inhibitor of P-gp, so an interaction with dabigatran can be expected. Serrao A. et al. have recently reported the successful use of the full dose of edoxaban in three HIV-infected patients with optimal tolerability during a 6- to 10-month follow-up [173]. No significant interactions are expected between NOACs and NRTIs, maraviroc, or fusion inhibitors (enfuviritide), but they should be used with caution [169].

A more recent analysis by Cattaneo D. and colleagues showed that only 14% of the HIV patients were administered NOACs, while the majority of patients still used VKA. According to those authors, the correct choice of NOACs, with the assessment of possible interactions individually for each patient, could theoretically reduce the number of unwanted interactions due to the application of anticoagulant and antiretroviral therapy [174].

### 5.8. Treatment of Patients with PE Who Have Epilepsy

The use of anticoagulant therapy in patients with epilepsy is complex due to possible interactions between the anticoagulant and antiepileptic therapy. Phenobarbital, phenytoin, carbamazepine and oxcarbazepine are established inducers of both metabolic enzymes such as CYP3A4 and/or P-gp [175]. Through the induction of enzymes, these drugs can affect the reduction of the concentration of NOACs in the plasma and thus reduce their clinical effectiveness [176]. According to European recommendations, these drugs are contraindicated for use with dabigatran and rivaroxaban, while they can be used with caution with apixaban and edoxaban, especially in the case of polypharmacy [141].

Valproate is able to inhibit the activity of CYP2C9, and, to a lesser extent, CYP3A4 and CYP2C19, as well as UGT1A4 and UGT2B7 [175]. The potential drug–drug interactions still remain unknown in other anti-epileptic drugs, such as pregabalin, gabapentin, lamotrigine, lacosamide, and topiramate, although individual cases of their interactions with NOACs are reported [153]. In a prospective cohort study in patients with non-valvular AF who were treated with NOACs and antiepileptics, a high frequency of thromboembolic events was demonstrated. Acton EK and colleagues examined the trend in the use of NOACs in relation to VKAs in the period from 2010 to 2018. The use of NOACs increased significantly in the period from 2010–2018 with a decrease in the use of VKAs. The importance of these results indicates the necessity of a better understanding of prescribing and managing anticoagulant therapy in patients with epilepsy due to possible drug–drug interactions [177].

### 5.9. Treatment of Chronic Thromboembolic Pulmonary Hypertension (CTEPH)

Chronic thromboembolic pulmonary hypertension (CTEPH) occurs in about 3% of patients who survive PE [178]. The exact pathophysiology of why CTEPH occurs in fewer patients after PE remains unknown. The following reasons are most often cited: a delay in diagnosis, presence of thrombosis in most branches of the pulmonary artery, recurrent symptomatic pulmonary embolism, and failure to apply thrombolytic therapy. A diagnosis of CTEPH is made by the presence of an elevated mean pulmonary artery pressure ≥ 25 mmHg and the presence of at least one segmental perfusion defect despite 3 months of anticoagulation therapy [179]. Bilateral pulmonary endarterectomy is a curative treatment for CTEPH, but life-long anticoagulant therapy is indicated in most patients.

## 6. Conclusions

Both the survival of patients with PE and their future quality of life depends on a quick and correct diagnosis of PE and an adequate therapeutic regimen.

The timely administration of thrombolytic therapy and/or embolectomy is life-saving in patients with high-risk pulmonary embolism. Most therapeutic doubts still exist in patients who are hemodynamically stable on admission and who have signs of right heart dysfunction on echocardiography and positive values of biomarkers, indicating both myocardial necrosis and right heart dysfunction. Within that group of patients, how do we single out those who are at risk of deterioration and who would benefit unequivocally from the initial application of thrombolytic therapy? For now, the current guidelines of the European Association of Cardiologists for PE do not single out such a group of patients but suggest the initial application of anticoagulant therapy. We are not sure that when hemodynamic destabilization occurs, the application of thrombolytic therapy will always be effective. Additionally, the adequate dose of thrombolytic therapy in patients with intermediate–high risk embolism is not clearly defined. Defining the dose is very important both for survival and for reducing the risk of resistant pulmonary hypertension, which is a permanent problem and requires the prolonged use of anticoagulant therapy. For the same reasons, it is necessary to introduce new studies and recommendations that will clearly define the initial therapeutic regimen in patients with intermediate-high risk pulmonary embolism.

In the treatment of PE, NOACs are recommended before VKA except in patients with antiphospholipid syndrome, mechanical valves, severe mitral stenosis and in pregnancy. Special attention should be paid to the interactions of NOACs with other drugs.

Our work clearly indicates that PE is a disease that we must think about and where therapeutic challenges still exist.

## Figures and Tables

**Table 1:** Treatment with anticoagulant therapy in patients with pulmonary embolism.

Drug	Dose	Special Consideration	Specific Patient Characteristics	Reversal Agents
**UFH**	80 unit/kg IV bolus, followed by an 18-unit/kg/h infusion;	**Avoid** HIT; Osteopenia Pronounced drag-drag interactions	Overt haemodynamic instability; (CrCl) ≤ 30 mL/min; Pragnancy; Severe obesity	Protamine sulfate
**LMWH**	1 mg/kg twice daily 1.5 mg/kg once daily	**Avoid** with severe renal impairment	Pragnancy; Obesity;	Protamine sulfate
**VKA**	**Warfarin** 5 mg/day once daily 4 mg/day once day-patients > 70 years	Cross the placenta-**contraindicated in** **pregnancy**	Antiphospholipid syndrome; Mechanical heart valves; Extremely reduced renal function; Severe mitral stenosis;	4F-PCC 4 or FFP
**Apiksaban**	10 mg twice daily for 7 days followed by 5 mg twice daily	**Avoid in** CrCl < 15 mL/min Severe hepatic impairment	Previous GI bleeding or high risk of bleeding; Patients with CA; Eldery patients;	Andexanet
**Rivaroxaban**	15 mg- twice daily (3 weeks) then 20 mg once daily (at least 6 months)	**Avoid in** CrCl < 30 mL/min; (FDA) CrCl < 15 mL/min (EMA).	Low risk of bleeding and without gastrointestinal tumours; Patient preference—a single dose regimen;	Andexanet
**Dabigatran**	150 mg—twice daily 110 mg—twice daily for patients ≥ 80 years	**Avoid in** CrCl < 30 mL/min.; Concomitant treatment with P-gp inhibitors in patients with CrCl < 50 mL/min; Reduce dose to 110 mg for patients ≥ 80 years or ≥75 years with at least one bleeding risk factor;	Can be removed by hemodialysis in patients with severe renal impairment;	Idarucizumab
**Edoxaban**	60 mg—once daily 30 mg—once daily if body weight ≤ 60 kg	**Avoid** CrCl < 15 mL/min. Severe hepatic dysfunction	Low risk of bleeding and without gastrointestinal tumours	Andexanet
**Fondaparinux**	5 mg subQ daily <50 kg 7.5 mg subQ daily—50–100 kg 10 mg subQ daily >100 kg	**Avoid** CrCl < 30 mL/min	HIT (off lable); alergy of LMWH	Factor VIIa
**Lepirudin**	0.4 mg/kg i.v over 15–20 min, followed by 0.15 mg/kg/h infusion	**Avoid** Severe renal impairtment	HIT	
**Argotroban**	2 μg/kg/min Liver dysfunction (bilirubin >1.5 mg/dL) → 0.5–1.2 μg/kg/min	**Avoid** in severe hepatic dysfunction	HIT	
**Bivalirudin**	0.15 mg/kg/h	**Avoid** Severe renal impairtment	HIT (off lable)	

Legend: **UFH**—unfractionated heparin; **LMWH**—low-molecular-weight heparin; **VKA**—vitamin K antagonists; **HIT**—heparin-induced thrombocytopenia; **CrCl**—creatinine clearance; **4F-PCC**—4-factor prothrombin complex concentrateor; **FFP**—fresh-frozen plasma; **GI**—gastrointestinal; **CA**—cancer; **P-gp inhibitors**—P glycoprotein inhibitors; **subQ**—subcutaneously.

## Data Availability

Data sharing not applicable.

## Supplementary Materials

The following supporting information can be downloaded at: https://www.mdpi.com/article/10.3390/ph15091146/s1, Figure S1: Transthoracic echocardiography - thrombus masses in both main branches of the pulmonary artery (RBPA-right branch of the pulmonary artery and LBPA -left branch of the pulmonary artery); Figure S2: Transthoracic echocardiography - thrombus masses in both main branches of the pulmonary artery and at the bifurcation of the main trunk; Figure S3: Transesophageal echocardiography of the pulmonary artery - thrombus mass in the right branch of the pulmonary artery; Figure S4: Transesophageal echocardiography -thrombus mass in the right branch of the PA (RPA); LPA-left branch of the PA; Video S1: Transesophageal echocardiography- thrombus mass in the right branch of the PA (RPA); LPA-left branch of the PA; Figure S5: Computed tomography of the pulmonary artery - thrombus masses in the left and right branches of the pulmonary artery; Figure S6: Computed tomography of the pulmonary artery - thrombus in both branches of the pulmonary artery and at the bifurcation of the main trunk PA; Figure S7: Computed tomography of the pulmonary artery - thrombus in both branches of the pulmonary artery and at the bifurcation of the main trunk.
