# Supplementary figures and images for: Dilemmas in the Choice of Adequate Therapeutic Treatment in Patients with Acute Pulmonary Embolism—From Modern Recommendations to Clinical Application
Source: Pharmaceuticals (Basel). 2022 Sep 14;15(9):1146. doi: 10.3390/ph15091146 (PMC9501350; doi:10.3390/ph15091146)

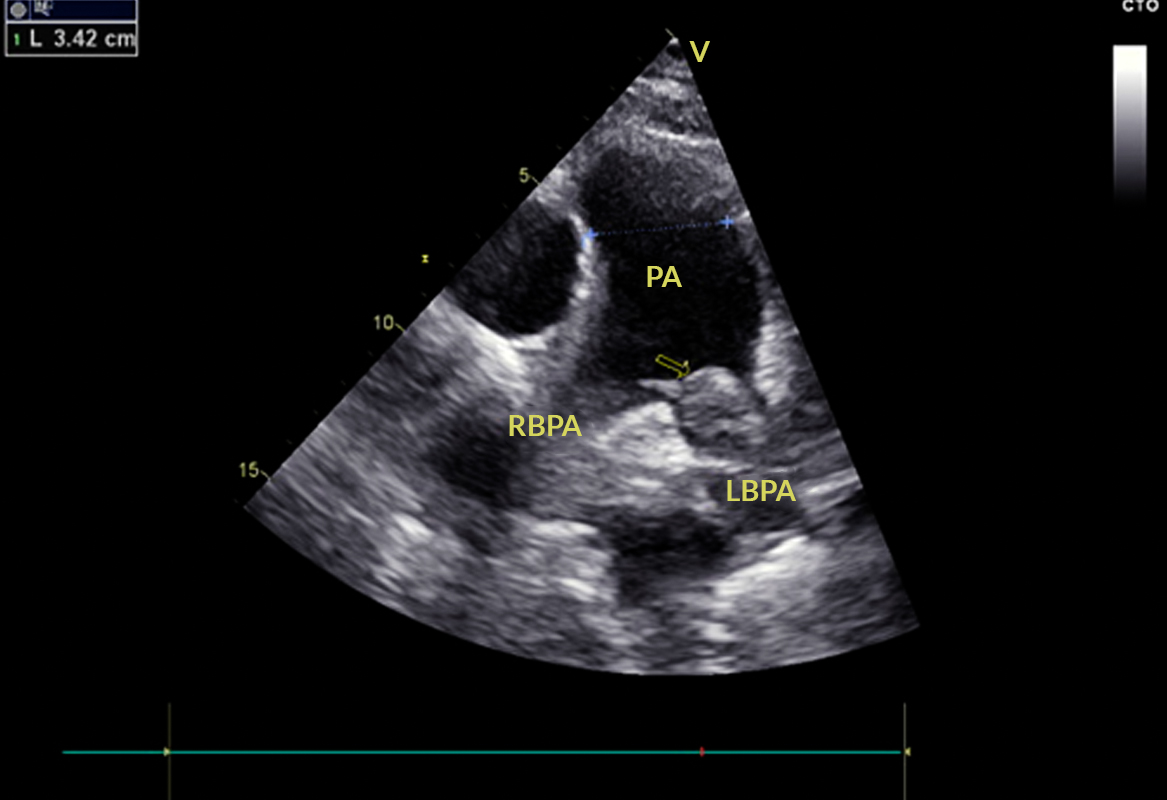

Supplement: Supplementary file 1 [file pharmaceuticals-15-01146-s001.zip › Figure S1. Transthoracic echocardiography - thrombus masses in both main branches of the pulmonary artery.jpg]

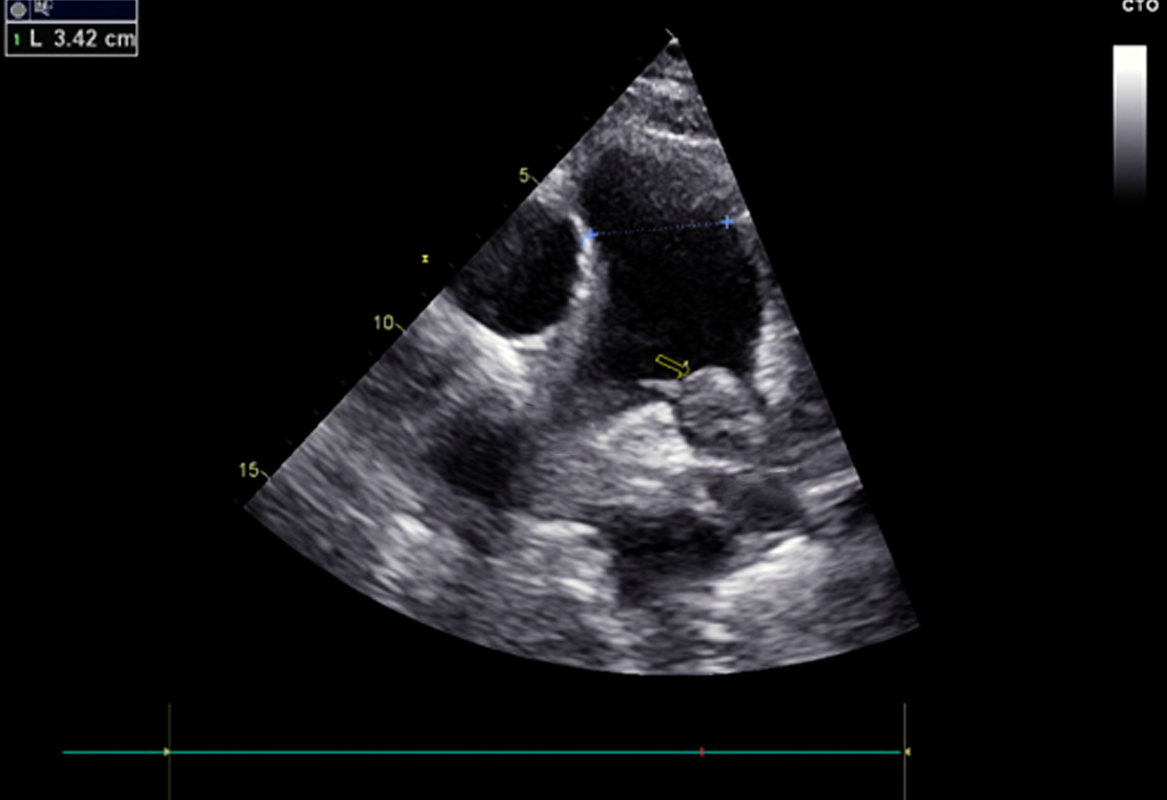

Supplement: Supplementary file 1 [file pharmaceuticals-15-01146-s001.zip › Figure S2. Transthoracic echocardiography - thrombus masses in both main branches of the pulmonary artery and at the bifurcation of the main trunk.jpg]

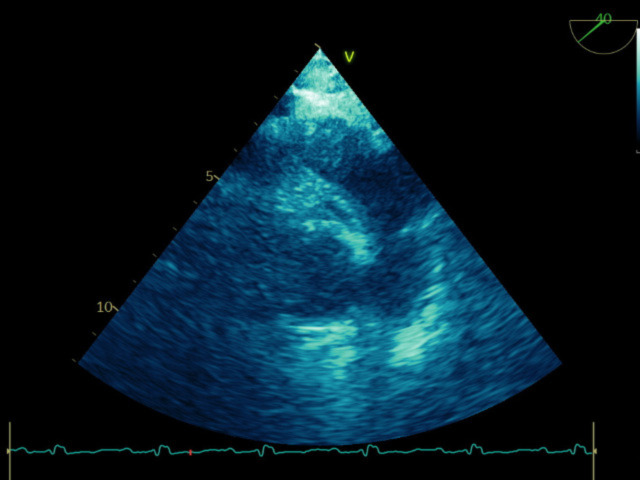

Supplement: Supplementary file 1 [file pharmaceuticals-15-01146-s001.zip › Figure S3. Transesophageal echocardiography of the pulmonary artery - thrombus mass in the right branch of the pulmonary artery.jpg]

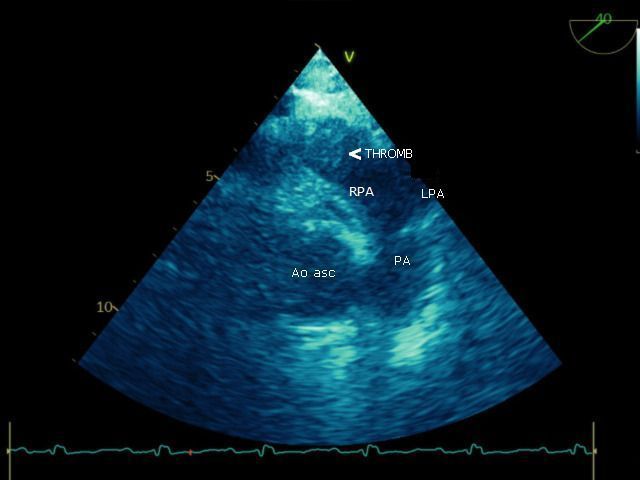

Supplement: Supplementary file 1 [file pharmaceuticals-15-01146-s001.zip › Figure S4. Transesophageal echocardiography -thrombus mass in the right branch of the PA (RPA); LPA-left branch of the PA.jpg]

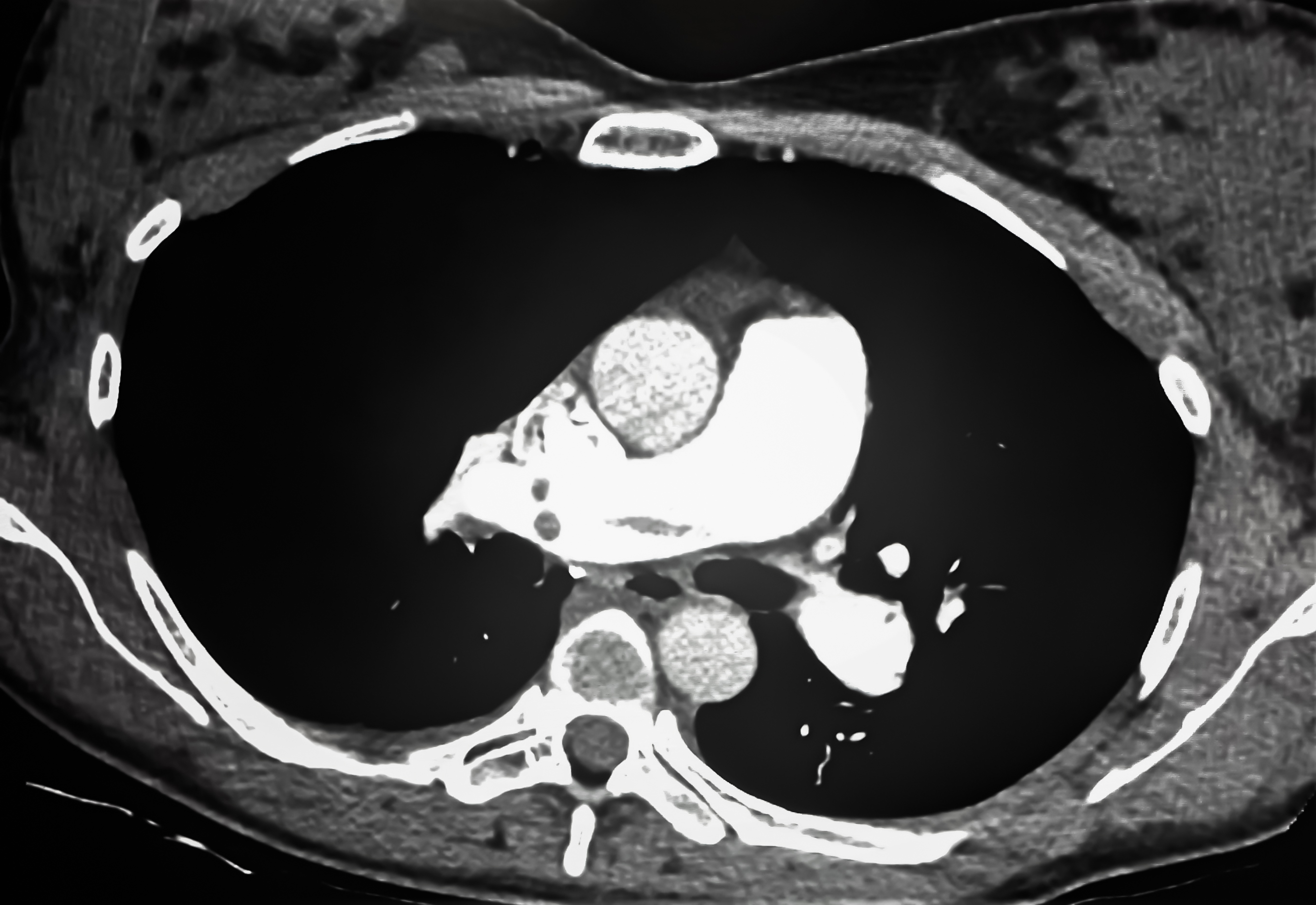

Supplement: Supplementary file 1 [file pharmaceuticals-15-01146-s001.zip › Figure S5. Computed tomography of the pulmonary artery - thrombus masses in the left and right branches of the pulmonary artery.jpg]

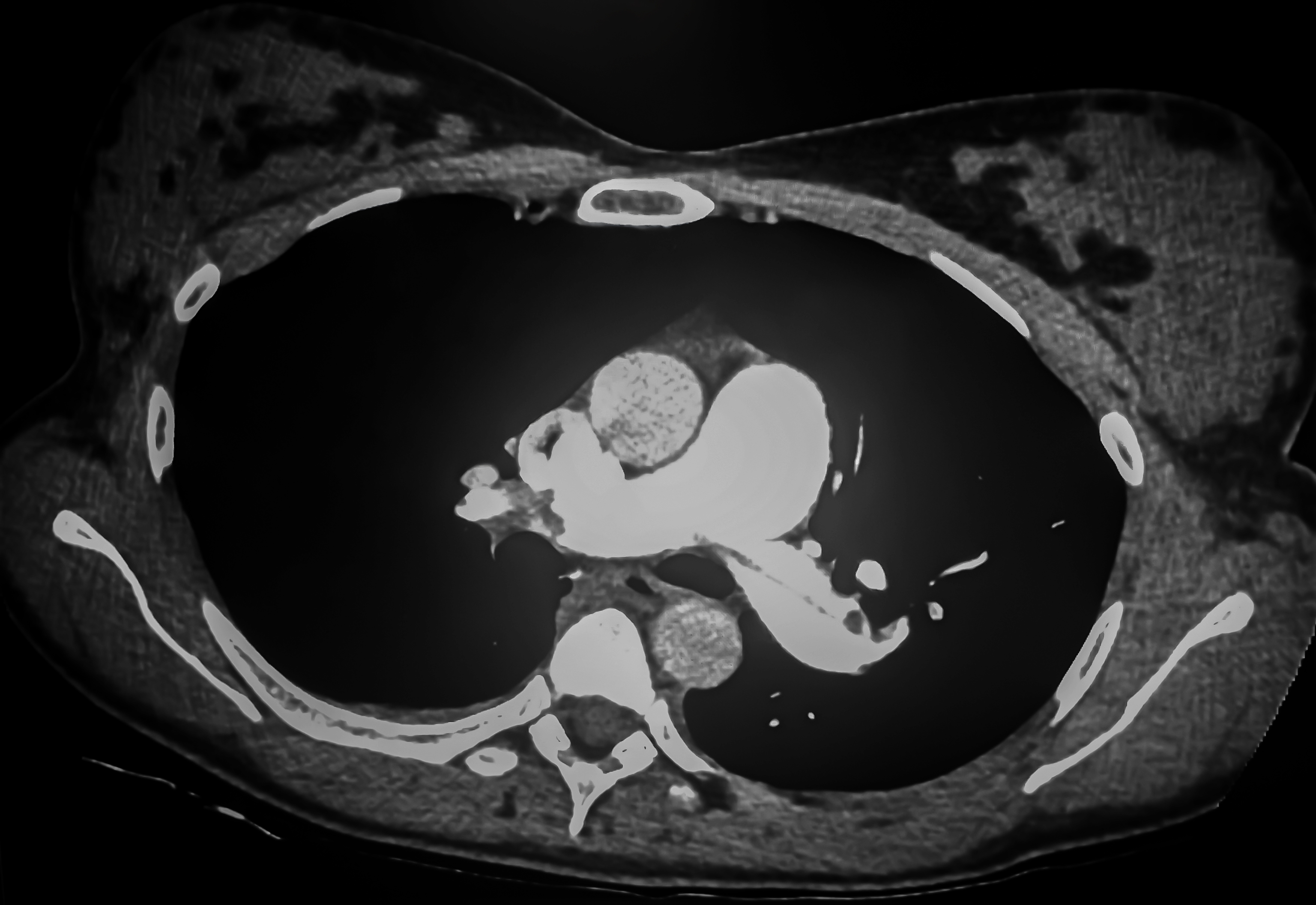

Supplement: Supplementary file 1 [file pharmaceuticals-15-01146-s001.zip › Figure S6. Computed tomography of the pulmonary artery - thrombus in both branches of the pulmonary artery and at the bifurcation of the main trunk PA.jpg]

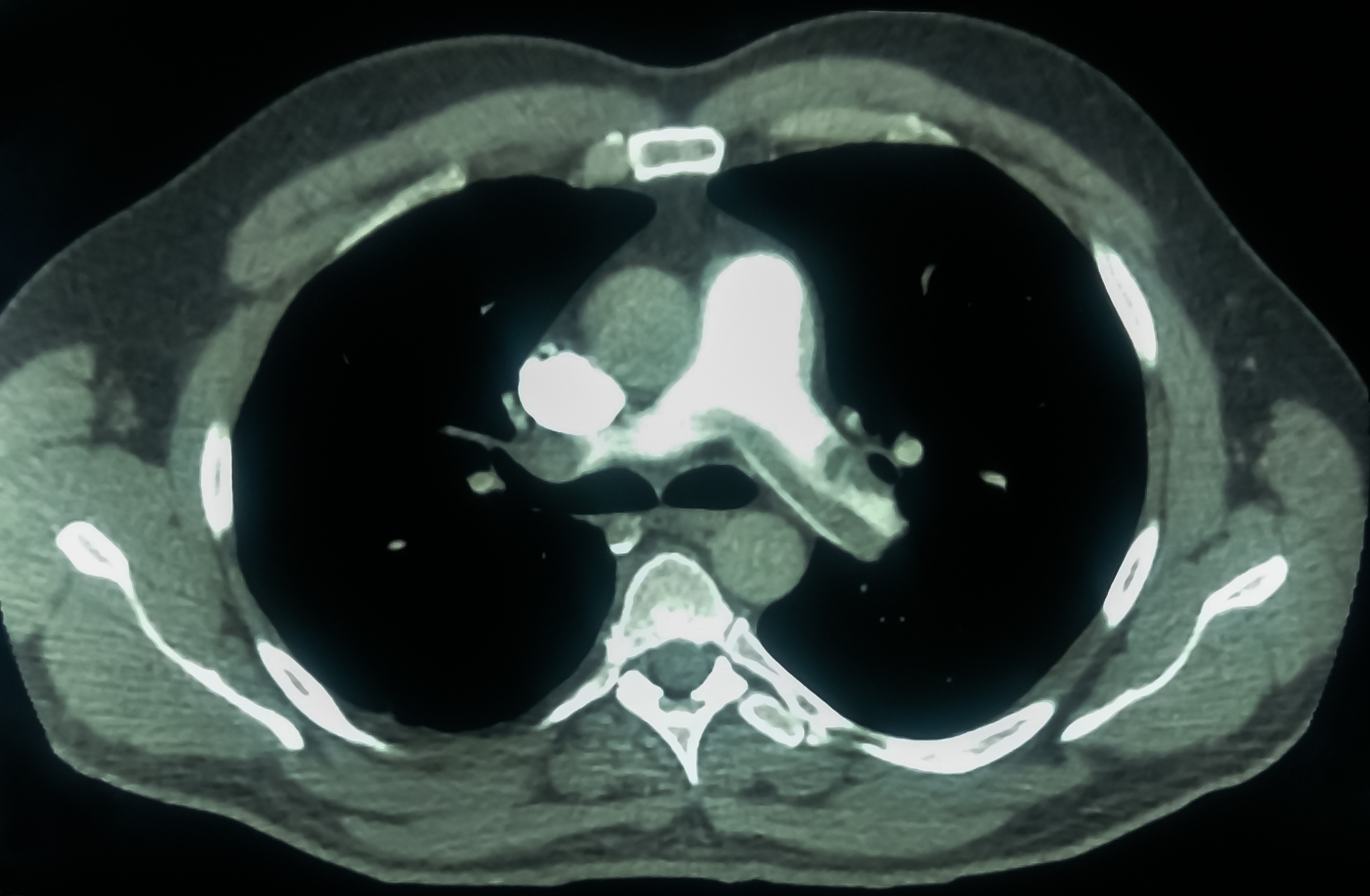

Supplement: Supplementary file 1 [file pharmaceuticals-15-01146-s001.zip › Figure S7. Computed tomography of the pulmonary artery - thrombus in both branches of the pulmonary artery and at the bifurcation of the main trunk.jpg]
